# Comparison of exosomes derived from induced pluripotent stem cells and mesenchymal stem cells as therapeutic nanoparticles for treatment of corneal epithelial defects

**DOI:** 10.18632/aging.103904

**Published:** 2020-10-13

**Authors:** Shudan Wang, Yunlong Hou, Xuran Li, Zhen Song, Baoqi Sun, Xinyue Li, Hong Zhang

**Affiliations:** 1 Department of Ophthalmology, The First Affiliated Hospital of Harbin Medical University, Harbin 150001, China; 2 College of Integrated Traditional Chinese and Western Medicine, Hebei University of Chinese Medicine, Shijiazhuang 050200, China; 3 National Key Laboratory of Collateral Disease Research and Innovative Chinese Medicine, Shijiazhuang 050200, China; 4 Department of Ophthalmology, Affiliated Hospital of Weifang Medical University, Weifang 261042, China

**Keywords:** induced pluripotent stem cells, mesenchymal stem cells, exosomes, corneal epithelial defect, therapeutics

## Abstract

Induced pluripotent stem cells and mesenchymal stem cells are pluripotent stem cells that represent promising therapies for treating various tissue injuries and wound healing. Exosomes are nanosized extracellular vesicles that have been identified as important mediators of therapeutic functions, which are performed via cell communication. In this study, we compared the efficacy of induced pluripotent stem cells-derived exosomes (iPSCs-Exos) and mesenchymal stem cells-derived exosomes (MSCs-Exos) in treating corneal epithelial defects. The characteristics of the two types of exosomes were not significantly different. Compared to MSCs-Exos, iPSCs-Exos had a better *in vitro* effect on the proliferation, migration, cell cycle promotion and apoptosis inhibition of human corneal epithelial cells. iPSCs/MSCs-Exos promoted cell regeneration by upregulating cyclin A and CDK2 to drive HCECs to enter the S phase from the G0/G1 phase. *In vivo* results from a corneal epithelial defect model showed that both iPSCs-Exos and MSCs-Exos accelerated corneal epithelium defect healing while the effects of iPSCs-Exos were much stronger than those of MSCs-Exos. This study demonstrated that iPSCs-Exos had a better therapeutic effect on corneal epithelial defect healing. Thus, a novel potential nanotherapeutic strategy for treating corneal epithelial defects and even more ocular surface disease could be undertaken by using iPSCs-Exos dissolved in eye drops.

## INTRODUCTION

The cornea is located on the surface of the anterior eye segment, serving as the first refractive element of the eye that focuses an image of the visual world for the retina [1]. Steady and rapid resurfacing of corneal epithelial defects is essential for preventing pathogen invasion and protect the underlying stroma [2]. Disruptions in the protective epithelial and stromal layers of the cornea can render the eye susceptible to infection, stromal ulceration, perforation, and scarring, which can lead to severe vision loss [3, 4]. Conventional management of corneal wounds consists mainly of supportive measures in the form of lubrication, antibiotics, and bandage contact lenses, and then an amniotic membrane for recalcitrant cases [5]. While there has been great progress in the treatment of corneal diseases, corneal defect healing in the setting of severe corneal disease or damage remains challenging [6]. In recent years, many preclinical studies have been carried out to investigate the application of stem cells for corneal defect healing [7].

Cell-based therapies are a recent development of wound healing technology, and they have attracted increasing attention and have the potential to replace conventional therapies [8]. Induced pluripotent stem cells (iPSCs) are a kind of stem cell that are generated by inducing the expression of transcription factors associated with pluripotency, allowing differentiated somatic cells to reverse their state to the embryonic stage [9], while mesenchymal stem cells (MSCs) are a type of multipotent stem cell and can be isolated from various adult or fetal tissues, including fat, bone marrow and umbilical cord blood [10]. Both iPSCs and MSCs are stem cells which have ability to undergo self-renewal and differentiate into every cell type in the body. iPSCs and MSCs have been studied as promising therapies for treating various tissue injuries; such therapeutic strategies include facilitating cutaneous wound healing, attenuating limb ischemia and enhancing bone regeneration [11–13]. iPSCs are promising for tissue engineering-based treatment of corneal epithelium damage and keratocyte dysfunction by differentiating into keratocyte-like cells [14]. Stem cell transplantation represents a very promising option for obtaining corneal epithelial cells and shows therapeutic equivalence with the hydrogel in the corneal epithelial wound healing [15]. Although stem cells have the potential for generating vast numbers of cells to replace damaged tissues, the potential risks and problems limit the possibility of clinical application, including tumor formation, ethical concerns, and graft rejection [16, 17]. About a third of ocular surface stem cell transplantation patients suffer immune rejection and the risk persists long term [18]. Proper handling of stem cells and the optimal storage conditions for maintaining cell viability and vitality are also challenges for transplantation [19].

Recent studies have provided evidence that the indirect interplay between donor cells and somatic cells occurs through the release of small vesicles, which contribute to creating an environment where maximum wound healing can be achieved, named exosomes. Exosomes are nanosized vesicles with a lipid bilayer and a size range of 30 to 200 nm, and they are released from most cell types via the plasma membrane; further, exosomes play pivotal roles in intercellular communication by transferring proteins and genetic information to target cells [15]. Due to the protective lipid bilayer membrane, exosomes can exist stably for a long time in the tissue microenvironment [20]. Exosomes contain complex molecular components, which include general and cell type specific lipids, proteins, mRNA, and microRNA, enabling them to function as vectors for multiple signaling among cells [21]. The important roles of exosomes have been shown in pathological conditions, such as inflammation, cancer, cardiovascular diseases, and diabetes, but they also have a role in wound healing [22–26]. Meanwhile, exosomes have been reported to play a major role in carrying out therapeutic functions via cell communication and modulating the molecular activities of recipient cells [27, 28]. Especially, the application of exosomes derived from stem cells has shown its superiority in maintaining similar functions and avoiding apparent adverse effects [29]. Therefore, exosomes likely have a great potential in cell repair, and several studies have demonstrated their role in cell protection and wound healing in cardiac, skin or skeletal muscle cells, for instance [30]. To the best of our knowledge, studies on the potential role of exosomes in corneal defects are limited; so too are studies on the differences in exosomes of stem cells from different sources. Samaeekia R et al reported that human corneal MSCs exosomes can accelerate corneal epithelial wound healing [5], however, the effect of iPSCs exosomes on corneal epithelial cells has not been investigated. Since iPSCs can be easily obtained without ethical constraints and can be expanded perpetually, they can be utilized as an optimal source of stem cells [31]. In view of the stable nature of iPSCs, we hypothesized that consistent iPSCs-derived exosomes (iPSCs-Exos) were a suitable target for mass production.

In the present study, we successfully isolated iPSCs-Exos and MSCs-derived exosomes (MSCs-Exos). Given the naturally healed-promoting properties of stem cells, we compared the effect of two types of exosomes in HCECs and investigated their therapeutic potential in a corneal epithelial defect model.

## RESULTS

### Characterization of iPSCs/MSCs-Exos

**Figure 1:**
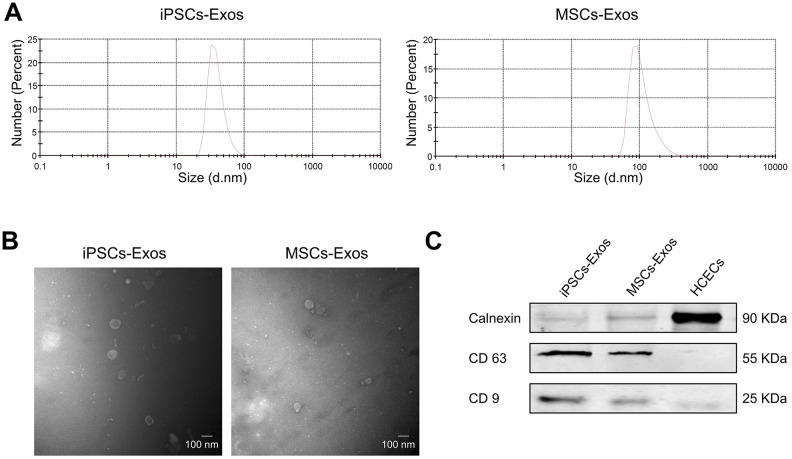
**Characterization of iPSCs/MSCs-Exos.** (**A**) NTA results demonstrate iPSCs/MSCs-Exos size distribution after isolation via ultracentrifugation. The diameter of iPSCs-Exos ranged from 30 to 120 nm while that of MSCs-Exos was between 60 and 400 nm. (**B**) TEM image of isolated exosomes showing a round morphology with diameters of approximately 100 nm. (**C**) Western blot illustrating the presence of the exosome markers CD9 and CD63, as well as the absence of the negative exosome markers Calnexin in isolated exosomes. HCEC cell lysate was used as a control.

We collected exosomes from the supernatant of iPSCs and MSCs by differential ultracentrifugation and evaluated their size distribution by nanoparticle tracking analysis (NTA). We found that size distribution of iPSCs-Exos ranged from 30 to 120 nm while it was 60-400 nm in MSCs-Exos (Figure 1A). Transmission electron microscopy (TEM) revealed that the exosomes were round-shaped with a size distribution consistent with what was observed by NTA (Figure 1B). There is no significant difference between the size and morphology of exosomes derived from iPSCs and MSCs. Western-blot analyses revealed that the iPSCs-Exos and MSCs-Exos expressed exosomal markers including CD9 and CD63, while the intracellular protein Calnexin (negative markers for exosomes) was absent in the isolated exosomes (Figure 1C).

### Uptake of iPSCs/MSCs-Exos by the corneal epithelium

**Figure 2:**
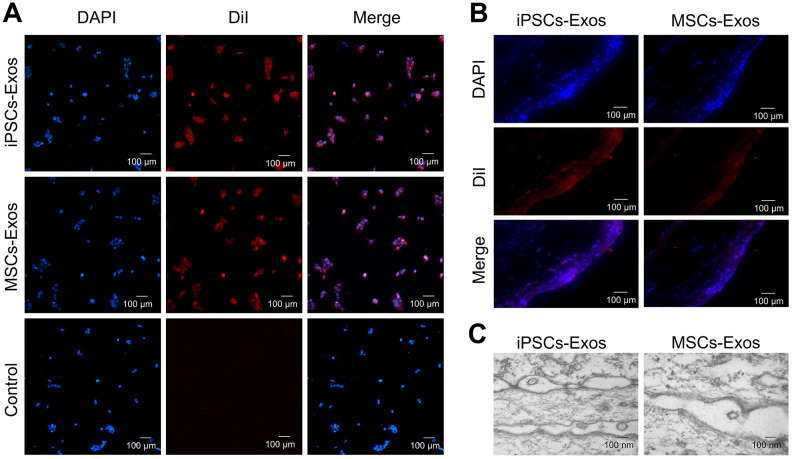
**Uptake of iPSCs/MSCs-Exos.** (**A**) Immunofluorescence staining of HCECs. HCECs were treated with 25 μg/ml DiI-labeled iPSCs/MSCs-Exos for 24 h. PBS, which was used to resuspend exosomes, was used as a negative control. Photomicrographs showed that red fluorescent particles were present throughout the cell cytoplasm, meaning that the exosomes were taken up by HCECs. (**B**) Immunofluorescence staining of rat corneal epithelium. After dropping DiI-labeled iPSCs/MSCs-Exos on rat cornea for 24 h, the corneas were harvested for immunofluorescence staining. The images of the whole mount of cornea showed a wide distribution of exosomes throughout the rat corneal epithelium, indicating successful fusion and uptake of iPSCs/MSCs-Exos by the corneal epithelium *in vivo*. (**C**) Exosome-like vesicles were detected by TEM on corneal epithelium. The diameters of the vesicles were between 100-200 nm.

To determine whether exosomes could be absorbed by HCECs, HCECs were incubated with 25 μg/ml DiI-labeled iPSCs/MSCs-derived exosomes for 24 h to optimize the internalization of exosomes by cultured cells. Photomicrographs showed that red fluorescent particles were present throughout the cell cytoplasm, meaning that the exosomes were taken up by HCECs (Figure 2A).

The uptake of exosomes was also verified *in vivo* following their topical application to the cornea of rats. After 24 h, whole mount imaging of the cornea showed a wide distribution of labeled exosomes throughout the mouse cornea indicating successful fusion and uptake of iPSCs/MSCs-Exos by the corneal epithelium *in vivo* (Figure 2B). There was no significant difference between iPSCs-Exos group and MSCs-Exos group in uptake. Exosomes and cell fusion were also verified by TEM. After adding drops of iPSCs/MSCs-Exos to the eyes of SD rats for 24 h, exosome-like vesicles were detected throughout the corneal epithelium. The diameters of vesicles were between 100-200 nm. We identified these vesicles as exosomes, rather than apoptotic bodies, based on their size (Figure 2C).

### Effects of iPSCs/MSCs-Exos on HCECs in vitro

The cell morphology of HCECs was analyzed after HCECs were stimulated with iPSCs/MSCs-Exos or DMEM/F12 medium for 48 h. HCECs with DMEM/F12 exhibited signs of atrophy while those with iPSCs/MSCs-Exos showed a fuller shape (Supplementary Figure 1).

**Figure 3:**
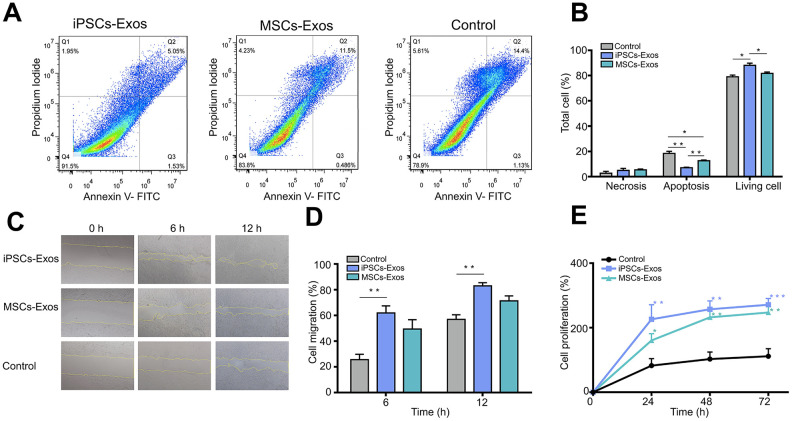
**Evaluation of the effect of iPSCs/MSCs-Exos on HCECs.** (**A**) HCECs were treated with 500 μg/ml iPSCs/MSCs-Exos or DMEM/F12 for 24 h and followed with apoptosis assay. (**B**) The percentage of cells at each stage is shown by the bar graphs. The iPSCs-Exos treatment group exhibited a lower proportion of apoptotic cells and a higher proportion of living cells than the control and MSCs-Exos treatment group. (**C**) HCECs were treated with 500 μg/ml iPSCs/MSCs-Exos for 12 h and followed with a scratch experiment. ImageJ was used to measure the cell migration area. (**D**) The cell migration rate was quantified at 6 and 12 h and is shown by bar graphs. The migration area was highest in iPSCs-Exos treated group at both 6 h and 12 h. (**E**) HCECs were treated with 500 μg/ml iPSCs/MSCs-Exos or DMEM/F12 for 72 h. Cell viability was detected by a CCK-8 kit and the results are shown with line graphs. iPSCs/MSCs-Exos increased cell viability at 24 h, 48 h and 72 h compared with that of the control. The data shown here are the mean ± SEM from three independent experiments. * P<0.05, ** P<0.01, *** P<0.001.

Annexin V-FITC/propidium iodide (PI) staining demonstrated the apoptotic rates of HCECs stimulated with iPSCs/MSCs-Exos (Figure 3A, 3B). The proportion of apoptotic cells was lowest in iPSCs-Exos group (7.027 ± 0.472%), HCECs apoptosis was less in MSCs-Exos group (12.65 ± 0.533%) than in control group (18.343 ± 1.732%).

Correspondingly, the proportion of living cell in iPSCs-Exos treated group is highest in three groups, which indicated that iPSC-Exos more strongly inhibited the apoptosis of HCECs.

In addition, to assess the effect of iPSCs/MSCs-Exos on corneal epithelial defect healing, a monolayer of confluent HCECs was scratched and then treated with 500 μg/ml iPSCs/MSCs-Exos for 12 h. Cell migration in monolayers treated with iPSCs-Exos was significantly accelerated, with 61.93 ± 5.487% migration area after 6 h, compared to that of the control which was 25.604 ± 4.212%. After 12 h, the migration area was 83.029 ± 2.454% in iPSCs-Exos group while it was 56.971 ± 3.531% in the control group. MSCs-Exos also increased the migration of HCECs (6 h: 49.467 ± 7.263%; 16 h: 71.374 ± 3.864%) compared with control (Figure 3C, 3D).

Moreover, we tested the cell viability of HCECs which showed a time-dependent growth trend in all groups. Treatment with iPSCs/MSCs-Exos increased cell viability at 24 h, 48 h and 72 h compared with the control. In each timepoint, cell proliferation was stronger in iPSCs-Exos group than that in MSCs-Exos group and was significantly different at 24 h (Figure 3E).

**Figure 4:**
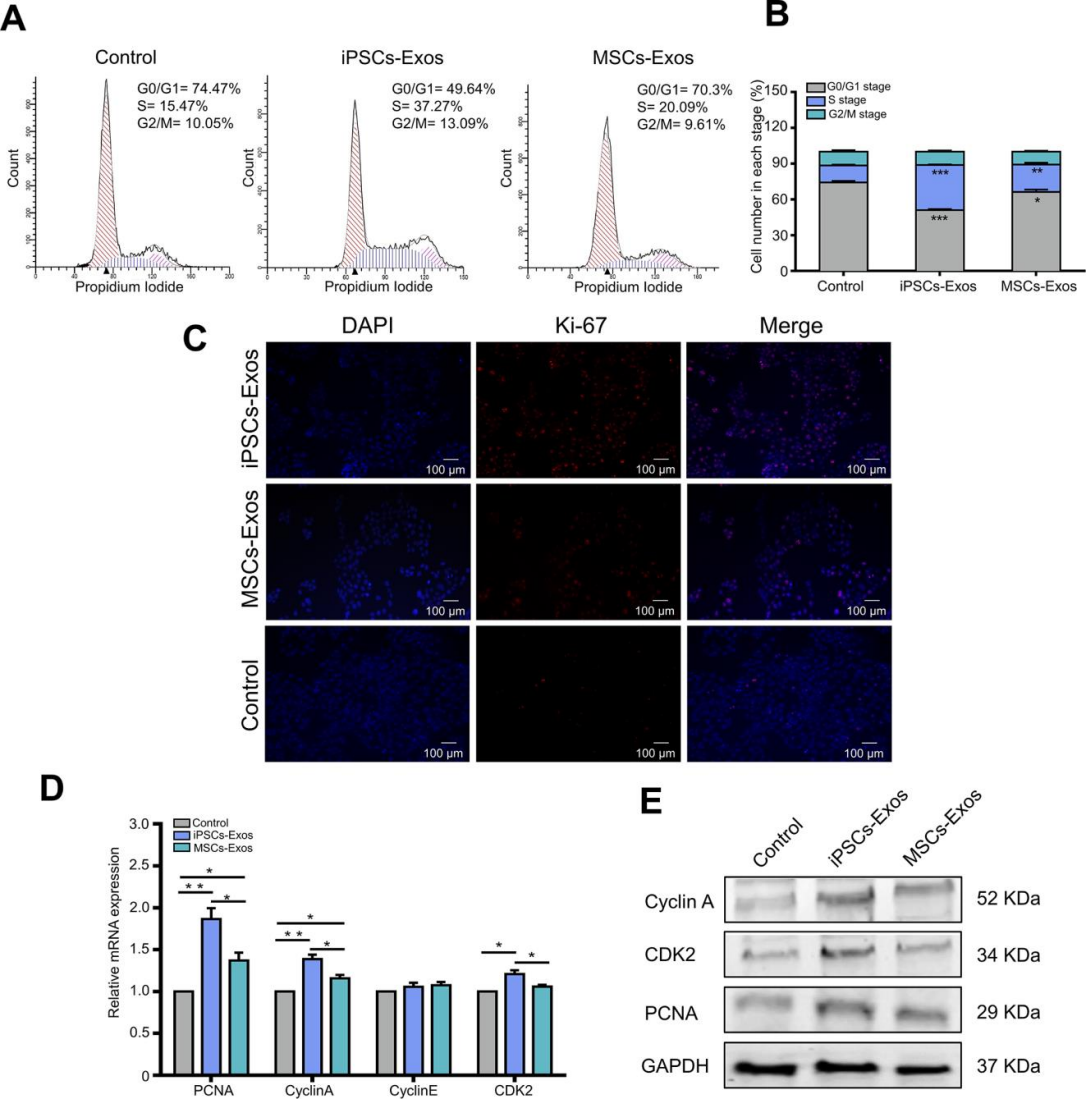
**Effects of iPSCs/MSCs-Exos on the cell cycle.** (**A**)HCECs were treated with iPSCs/MSCs-Exos. Then, after 24 h, the distribution of cell cycle was determined by flow cytometry. (**B**) The cell number in each stage was quantified and is shown in the bar graphs. iPSCs-Exos significantly increased the number of HCECs in the S phase, which was accompanied by a significant decrease in the number of cells in the G0/G1 phase. (**C**) After HCECs were stimulated with iPSCs/MSCs-Exos for 48 h, immunofluorescent staining was performed with anti-Ki-67 antibody. The nuclei were stained by DAPI. (**D**) Quantitative RT-PCR analysis of HCECs showed that PCNA, cyclin A and CDK2 mRNA were upregulated in iPSCs-Exos group compared with MSCs-Exos group and the control. (**E**) Protein levels of PCNA, cyclin A and CDK2 were measured by western-blot after treatment with iPSCs/MSCs-Exos for 24 h. The data shown here are the mean ± SEM from three independent experiments. * P<0.05, ** P<0.01.

As the cell cycle is one of the major reasons for cell growth, then we further explored the effect of iPSCs/MSCs-Exos on cell cycle distribution of HCECs. Cells were treated with PI to stain DNA then sorted for PI levels by flow cytometry, generating a profile of the cell population indicative of their cell cycle stage. Treatment with 500 μg/ml iPSCs/MSCs-Exos for 24 h significantly increased the number of HCECs in the S phase, which was accompanied by a significant decrease in the number of cells in the G0/G1 phase (Figure 4A, 4B). Moreover, the proportion of cells in the S phase was higher in iPSCs-Exos group than it was in MSCs-Exos group. The results indicated that iPSCs-Exos promoted the cell cycle progression of HCECs, driving HCECs to exit the G0/G1 phase and enter the S phase.

It is evident that iPSCs/MSCs-Exos may induce proliferation of HCECs *in vitro* from the above results. To identify whether iPSCs/MSCs-Exos is strictly associated with cell proliferation on the molecular level, we used Ki-67 as a marker to determine the growth fraction of HCECs population. As shown in Figure 4C, nuclear protein Ki-67 was positively stained in HCECs and the fluorescence intensity was strongest in iPSCs-Exos group.

To determine which molecules participate in the regulation of iPSCs/MSCs-Exos on HCECs proliferation and cell cycle, HCECs treated with iPSCs/MSCs-Exos were subjected to qRT-PCR and western-blot analysis. As a result, we found that genes associated with HCECs proliferation marker PCNA were significantly increased at mRNA level within 24 h of exposure to 500 μg/ml iPSCs/MSCs-Exos. In addition, cyclin A and CDK2 which play important roles in the cell cycle, were robustly upregulated in response to iPSCs-Exos stimulation (Figure 4D), providing a molecular basis for the flow cytology results, while cyclin E showed no significant difference among the three groups. The protein levels of PCNA, cyclin A and CDK2 were consistent in terms of gene expression (Figure 4E).

### iPSCs/MSCs-Exos accelerate corneal epithelial defect healing *in vivo*

**Figure 5:**
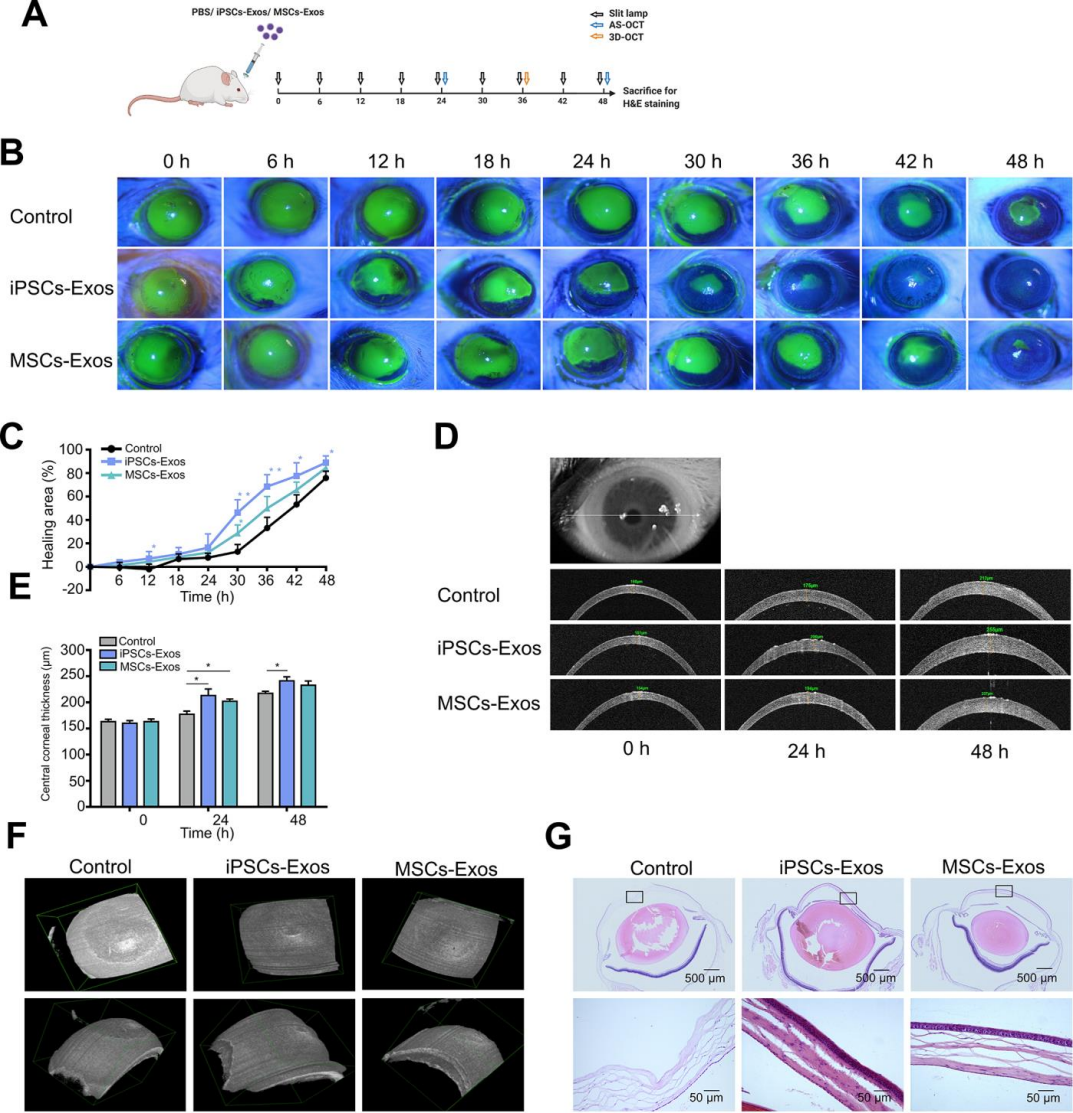
**Effect of iPSCs/MSCs-Exos on corneal epithelial defect healing in vivo.** (**A**) Schematic diagram of the experimental procedure. After corneal epithelial defect and treated with iPSCs/MSCs-Exos every 6 h, different procedures were performed at relative time point. (**B**) The corneal epithelial defect area was monitored every 6 hours with fluorescein staining and slit lamp. (**C**) The corneal epithelial defect healing rates are shown in line graphs. The cornea healing area in iPSCs-Exos group increased obviously compared with that in the vehicle group and was statistically significant at 12, 30, 36, 42, and 48 h compared with control group. (**D**) Central corneal thickness was measured by AS-OCT at both 24 and 48 h. (**E**) Central corneal thickness is shown by bar graph. Central corneal thickness in the iPSCs-Exos group was statistically different from that of the control. (**F**) A 3D-pattern was generated by AS-OCT for rat corneas at 36 h, enabling a more stereoscopic view of the corneal epithelial defects. (**G**) Rat eyeballs were harvested 48 h after corneal defect and were stained with hematoxylin and eosin. Data are representative of one of three independent experiments performed. Each experiment consisted of 4 animals/group. The values are shown as the mean ± SEM. * P<0.05, ** P<0.01.

Finally, we attempted to investigate the therapeutic potential of the iPSCs/MSCs-Exos for corneal epithelial defects *in vivo* using a mechanical injury model. iPSCs/MSCs-Exos or PBS were applied topically on the cornea following 2 mm epithelial debridement defect. Then, we monitored the defect of cornea for 48 h, with corresponding eye examinations at different time points (Figure 5A). We found that the defect area in iPSCs-Exos group decreased obviously compared with that in PBS group and had statistical significance at 12 h, 30 h, 36 h, 42 h and 48 h while the MSCs-Exos group had statistical significance only at 30 h. At 48 h, the healing area was 88.88 ± 2.925%, 84.38 ± 2.26% and 75.85 ± 2.875% in iPSCs-Exos group, MSCs-Exos group and control group respectively (Figure 5B, 5C).

Anterior segment optical coherence tomography (AS-OCT) scans were used to reveal the thickness of corneas during defect healing. The corneal thickness was restored after the removal of corneal epithelium. By measuring the central corneal thickness, we found that the corneas treated with iPSCs-Exos or MSCs-Exos were generally thicker than those in the vehicle group. The difference in central corneal thickness between iPSCs-Exos group and the vehicle group showed statistical significance at both 24 h and 48 h. At 48 h, the central corneal thickness was 241 ± 7.81 μm, 232.7 ± 8.11 μm and 217 ± 4.223 μm in iPSCs-Exos group, MSCs-Exos group and control group respectively (Figure 5D, 5E). With a 3D-pattern of OCT, the corneal epithelial defects were visualized stereoscopically after corneal epithelium deprivation for 36 h, the size and depth of corneal defects was smallest in iPSCs-Exos group (Figure 5F). The results demonstrated that iPSCs-Exos had a better effect on the restoration of corneal thickness after epithelium damage.

Hematoxylin and eosin (H&E) staining revealed the epithelial cell layers of the cornea. In PBS group, there were still some corneal epithelial defects in the central cornea, and 3-4 layers of corneal epithelial cells appeared in the peripheral cornea. In iPSCs/MSCs-Exos group, the corneal epithelium were integrated with 4-5 layers cells in MSCs-Exos group and 4-6 layers cells in iPSCs-Exos group (Figure 5G). This matched with the results of corneal thickness measured by AS-OCT.

## DISCUSSION

Stem cell-derived exosomes have demonstrated great ability to exert therapeutic effects in several diseases [16, 28]. Studies on the use of exosomes for corneal injury treatment have thus far been limited, and to date, no study has investigated the use of exosomes from iPSCs for corneal injury. To the best of our knowledge, this is the first demonstration of the therapeutic use of exosomes from two types of stem cells in corneal epithelial defect healing. Here, we compared the effect of iPSCs-Exos and MSCs-Exos in treating corneal epithelial defects. The ability of stem cell-derived exosomes to carry proteins and nucleic acid to travel between cells makes them an appealing cell-free therapy for multiple diseases. It has been shown that corneal MSCs exosomes can accelerate corneal epithelial defect healing [5]. Our results proved the effect of MSCs-Exos and demonstrated that iPSCs-Exos were stronger than MSCs-Exos in corneal epithelial defect healing.

In our study, after validating exosomes from iPSCs and MSCs through the classic methods NTA, TEM and western-blot, we confirmed the integration of iPSCs/MSCs-Exos to HCECs and corneal epithelium. According to the size distribution as well as the expression of characteristic markers, the isolated nanoparticles derived from iPSCs and MSCs were primarily exosomes. Then we systematically investigate the effect of iPSCs/MSCs-Exos on HCECs, including cell proliferation, migration, cell cycle and apoptosis *in vitro*. We found that the application of both iPSCs-Exos and MSCs-Exos application could increase HCECs migration and proliferation. Likewise, the anti-apoptotic effects of iPSCs/MSCs-Exos also suggested their potential to inhibit cell death caused by multiple injuries. In addition, iPSCs-Exos had a stronger effect on corneal epithelial cell proliferation, migration and inhibition of apoptosis than the same concentration of MSCs-Exos. In general, the optimal way in tissue repair could be to encourage the remaining cells to reenter the cell cycle to proliferate and replace dead cells. Flow cytometric analysis revealed that iPSCs/MSCs-Exos increased the proportion of cells in the S phase and decreased the proportion of cells in the G0/G1 phase, which meant that exosomes derived from stem cells promoted cell cycle progression of HCECs. Cell cycle progression is regulated by cyclins and cyclin-dependent kinases (CDKs) [32]; specifically, cyclin D/CDK4 or CDK6 regulate the progression of the G1 phase, and cyclin E/CDK2 and cyclin A/CDK2 facilitate the G1 to S-phase transition [33, 34]. Our results showed that HCECs treated with iPSCs/MSCs-Exos promoted HCECs cell cycle progression mainly through upregulating cyclin A and CDK2. In our study, we applied two exosomes in a same concentration to compare their effects on HCECs and found that iPSCs-Exos showed stronger effects in all aspects we investigated than MSCs-Exos. Notably, a recent report showed that iPSCs-Exos shared 76.63% of proteins with iPSCs while MSCs-Exos shared only 37.32% of the proteins with MSCs, so the richer protein content might have broader cellular functions in the application of iPSCs-Exos [35].

Then, to compare the efficacy of iPSCs/MSCs-Exos in corneal epithelial defects *in vivo*, we established a rat epithelial mechanical injury model and monitored for 48 h. Corneal examinations and eyeball H&E staining revealed that both iPSCs-Exos and MSCs-Exos accelerated the corneal epithelial defect healing while iPSCs-Exos did better than MSCs-Exos. 3D-OCT has been used for detecting lens position, macular and optic nerve head in the eye [36–38]. However, the application of 3D-OCT to the cornea has not been reported. Here we applied 3D-OCT on cornea to have a more stereoscopic perspective of cornea epithelial defect morphology. Corneal epithelium is maintained by peripherally located limbal stem cells that produce transient amplifying cells which proliferate, migrate centripetally, differentiate and are eventually shed from epithelial surface [39]. Thus, we preserved peripheral cornea and found corneal epithelium grew from periphery to center. Fluorescein staining, 3D-OCT and H&E results demonstrated that there existed a vacancy in corneal center in control group while corneal epithelium is almost integral in iPSCs/MSCs-Exos group.

**Figure 6:**
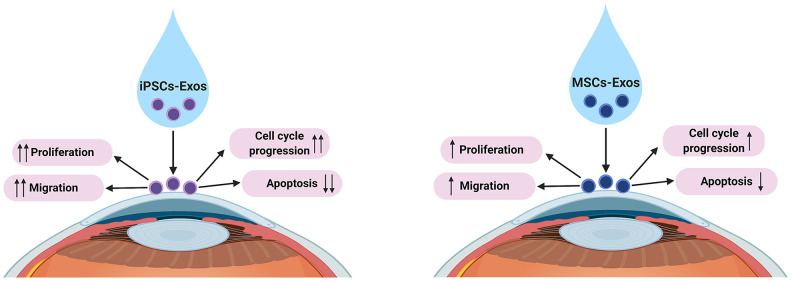
**Proposed underlying mechanisms of iPSCs/MSCs-Exos in corneal epithelial defect healing.** We propose that both iPSCs-Exos and MSCs-Exos can promote corneal epithelial repair and regeneration by enhancing HCECs proliferation, migration, promoting cell cycle development and attenuating apoptosis. iPSCs-Exos showed stronger effect on HCECs than MSCs-Exos in all aspects.

In summary, we demonstrated that both iPSCs-Exos and MSCs-Exos absorbed by corneal epithelial cells could promote corneal epithelial defect healing mainly by regulating cell metabolism. iPSCs-Exos produced better effects than MSCs-Exos on processes such as cell proliferation, migration, cell cycle regulation and inhibition of apoptosis (Figure 6). In general, *in vivo* and *in vitro* experiments showed that iPSCs-Exos are superior in corneal re-epithelialization. Exosomes have several advantages over the delivery of stem cells to the injury site. The bi-lipid membrane of exosomes can maintain the encapsulated proteins, mRNA and microRNA under stable conditions and enable a long-lasting effect [40]. As exosomes have excellent stable chemical properties and high biocompatibility, they can promote wound repair without the risk of immunological rejection and can be safely stored [41]. Therefore, topical application of iPSCs-Exos brings a new perspective on corneal epithelial cell injuries. Further investigations into the active factors within iPSCs-Exos are necessary to elucidate their mechanisms of action.

## CONCLUSIONS

In this study, we demonstrated that iPSCs-Exos had a stronger therapeutic effect on corneal epithelial defects than MSCs-Exos. As exosomes are nanomaterials that can be dissolved to form eye drops for the treatment of eye diseases, our findings may provide a novel potential therapeutic tool, iPSCs-Exos, for corneal epithelial defects and additional ocular surface diseases.

## MATERIALS AND METHODS

### Preparation of exosomes

Undifferentiated iPSC lines were maintained on Matrigel (Stemcell, US) treated six-well plates in PGM1 medium (Cellapy, China) at 37°C in a humidified incubator with 5% CO_2_. Cells between passages 30 and 50 were used for exosomes collection. Human umbilical cord-derived MSCs were harvested and cultured as reported previously [42]. In brief, MSCs were cultured in mesenchymal stem cell serum-free medium (Yocon, China). MSCs between passages 3 and 7 were used for the subsequent experiments. HCECs were maintained in complete DMEM/F12 with 10% fetal bovine serum (HyClone, Australia).

Exosomes were isolated as previously described with minor modification [27]. Briefly, conditioned medium samples were centrifuged at 300 g for 10 min followed by 1500 g for 10 min at 4°C to remove cells and cellular debris. Next, the supernatant was filtered through a 0.22 μm bacterial filter (Corning Incorporated, US) for further purification of exosomes, and then the filtrate was ultracentrifuged at 110,000 g for 70 min at 4°C to pellet exosomes. The exosomes were then washed in a large volume of phosphate-buffered saline (PBS) and ultracentrifuged again at the same high speed for 70 min. The pelleted exosomes were resuspended in PBS.

### Validation of exosomes

For subsequent TEM examination, exosome samples were prepared by a routine “negative staining” method. Exosomes were directly adsorbed onto Formvar-carbon-coated 400 mesh copper EM grids (Pelco, US) and were negatively stained using 2% aqueous phosphotungstic acid (PTA), then, they were air dried, prior to examination by JEM-1220 TEM (JEOL, Japan), operating at an accelerating voltage of 80 kv. To measure the size of exosomes, NTA was performed using the Nanosight system (Malvern Zetasizer, England) as described previously [43]. The capture settings and analysis settings were established manually according to the manufacturer’s instructions. Exosomal surface marker proteins CD63 and CD9 and the negative marker Calnexin were analyzed by western-blot [44]. HCECs were used as a negative control.

### Exosome uptake

Cellular uptake of exosomes was assessed by confocal microcopy [45]. iPSCs-Exos and MSCs-Exos were labeled with DiI fluorescent dye (1,1′-dioctadecyl-3,3,3′,3′-tetramethylindocarbocyanine perchlorate; Beyotime, China), which labels the plasma membrane. Briefly, exosomes were incubated with DiI dye for 1 h in the dark. Then labeled-exosomes were resuspended in 4 ml PBS followed by ultracentrifugation at 110,000 g for 1 h. The pellets were resuspended in 100 μl of PBS. HCECs were incubated with labeled exosomes for 24 h and then washed with PBS to remove unbound exosomes. Mounting medium with 4',6-diamidino-2-phenylindole (DAPI; Vector Laboratories, US) was used to stain nuclei, and images were captured under a confocal microscope (Carl Zeiss, Germany).

To examine the uptake of iPSCs/MSCs-Exos by the corneal epithelium *in vivo*, DiI-stained exosomes were applied topically to the cornea of rats, and assessment occured 24 h later. The cornea was then obtained from rats and stained with DAPI, mounted on a slide, and imaged with confocal microscopy. The corneal epithelium of rats was also analyzed by TEM with the same method described above.

### Corneal epithelial defect model

Corneal defect experiments in rats were conducted in compliance with the ARVO Statement for the Use of Animals in Ophthalmic and Vision Research. The protocol was approved by the Committee on the Ethics of Animal Experiments of the First Affiliated Hospital of Harbin Medical University. Six-week-old male SD rats were anesthetized by an intraperitoneal injection of with a mixture of ketamine (75 mg/kg) and xylazine (13.5 mg/kg) with only one eye treated under topical anesthesia (0.5% proparacaine). A circle defect (2 mm in diameter) was produced in the corneal epithelium of SD rats by an AlgerBrush II (The Alger Company, US) and reaver blade as previously described [46]. The wounded corneas were then treated topically every 6 h with 5 μl of exosome suspension, which contained 500 μg/ml iPSCs/MSCs-Exos or PBS. Defect closure was monitored with fluorescein staining and photographed with a camera-equipped slit lamp every 6 h until recovery. The percentages of defect closure were calculated based on the baseline for each mouse using ImageJ software (Rewak Software, Germany).

AS-OCT (Optovue, USA) was applied to rats at baseline, 24 h and 48 h after treatment to measure the variation in corneal thickness. Once anesthetized, rats were placed on the animal imaging mount. Horizontal lines scans through the corneal vertex were carried out on central fixation to show sections across the central and peripheral cornea. Corneal thickness was measured for the central region [47]. Thickness (μm) was measured as the distance from epithelium to endothelium, at the point where a vertical line was orthogonal to the anterior corneal curvature. For a better perspective of cornea epithelial wound morphology, each wound pattern was measured by rotating the 3D-reconstructed images for the central regions at 36 h.

### Hematoxylin and eosin staining

The eyeballs harvested from rats after they were sacrificed were immediately fixed in 4% paraformaldehyde (PFA, Beyotime, China) overnight. The fixed samples were embedded in paraffin for performing the histological analysis. 6 μm thick sections were stained with H&E and examined under a light microscope. The whole cornea and the local corneal epithelium were observed under 4 × magnification and 40 × magnification, respectively.

### Cell proliferation assay

To evaluate the proliferative capacity of HCECs treated with exosomes, HCECs were seeded at 1x10^4^ per well in 96-well culture plates. iPSCs/MSCs-Exos were added at a concentration of 500 μg/ml to the DMEM/F12 culture medium after the cells were attached. Untreated HCECs exposed to the same volume of culture medium served as a normal control. A total of 10 μl of Cell Counting Kit-8 (CCK-8; Beyotime, China) reagent was added after iPSCs-Exos treatment. Following a 4 h incubation, the plate was measured at 450 nm with a spectrophotometer (Bio-Rad, US).

### Cell migration assay

A wound healing assay was performed for the migration of HCECs. HCECs were seeded into 12-well plates and grown until they reached confluence. A sterile 200-μl pipette tip was used to scratch the cell layers. After washing with PBS for three times, the culture medium was replaced with DMEM/F12 basal medium containing 500 μg/ml iPSCs/MSCs-Exos. Control dishes were similarly scratched and cultured in the same volume of culture medium. After 6 h and 12 h of adding exosomes, the cells were photographed. The distance of cell migration was measured using ImageJ software.

### Evaluation of apoptosis

To compare the apoptosis of HCECs under different treatments, 0.5×10^6^ cells were stimulated with iPSCs/MSCs-Exos at a concentration of 500 μg/ml. After 48 h, cells were harvested by transferring them with complete medium into 1.5 ml reaction tubes. Cells were centrifuged at 500 g for 5 min at 4°C and the supernatant was discarded. Cells were washed with 1 ml PBS and resuspended in 100 μl of staining buffer (Annexin V-FITC/PI Apoptosis Detection Kit, Meilunbio, China); then, they were mixed with 5 μl of Annexin-V-FITC and 10 μl of PI in incubation buffer according to the manufacturer's instructions. The cells were incubated for 15 min at room temperature and were protected from light. After centrifugation at 500 g for 5 min at 4°C, cells were resuspended in 100 μl PBS and analyzed by a flow cytometer (Beckman Coulter, US).

### Cell cycle assay

Flow cytometry was also used to evaluate the cell cycle of HCECs. HCECs (1×10^6^) that were untreated or were treated with 500 μg/ml iPSCs/MSCs-Exos were harvested, washed with PBS, and fixed in 75% ethanol overnight at 4°C. Prior to analysis, cells were washed again with PBS, resuspended and treated with 0.25 mg/ml ribonuclease A (Cell Cycle and Apoptosis Analysis Kit, Meilunbio, China) for 30 min at 37°C. Then, they were incubated with 20 mg/ml ribonuclease A in the dark for 30 min. Finally, the samples were immediately analyzed.

### Quantitative real-time polymerase chain reaction (qRT- PCR) analysis

Total RNA was extracted using TRIzol reagent (Invitrogen, China) according to the manufacturer’s instructions. The isolated RNA was reverse-transcribed to generate synthesize cDNA in a 20 μl reaction mixture. The following RT-PCR conditions were used: 15 min at 95°C, followed by 40 cycles for 10 s at 95°C, 30 s at 60°C, and 1 s at 72°C,; then, there was 1 cycle of cooling for 30 s at 50°C. The primers were synthesized by Co Invitrogen Ltd. (China). The sequences of the primers are shown in Supplementary Table 1. Glyceraldehyde 3-phosphate dehydrogenase (GAPDH) was used as a control.

### Western-blot assay

Protein extraction and western-blot analysis were performed according to a previously described protocol [16]. In brief, treated cells or exosomes were homogenized in RIPA buffer with protease inhibitor. The protein level was determined using a BCA protein assay kit (Beyotime, China). Samples were run on 12% SDS-PAGE gels, blotted onto PVDF membranes, and then blocked with 5% nonfat dried milk for 2 h at room temperature Afterward, the PVDF membranes were incubated overnight at 4°C with the following antibodies: mouse anti-Cyclin A1+Cyclin A2, anti-CDK2, anti-PCNA (Abcam, US) and rabbit anti-GAPDH (Cell Signaling Technology, US). The membranes were then rinsed five times with Tris-buffered saline containing 0.5% Tween-20, and were then incubated with goat anti-rabbit or anti-mouse IgG secondary antibodies (Cell Signaling Technology, US) for 1 h at room temperature. An Odyssey fluorescent scanning system (LI-COR, US) and Quantity One software were used to detect the immunoreactive proteins.

### Immunofluorescence staining

HCECs were untreated or were treated with 500 μg/ml iPSCs/MSCs-Exos for 48 h. Cultured HCECs grown on coverslides were fixed in 4% PFA, permeabilized via 0.1% Triton X-100 (Sigma-Aldrich, US) for 4 min, then washed three times in PBS. Cells were blocked with 5% goat serum (Sigma-Aldrich, US) for 1 h and incubated with mouse anti-Ki67 antibody (Abcam, US) overnight at 4°C. The Cy^TM^3-conjugated anti-mouse IgG antibody (Jackson ImmunoResearch, US) was used as a secondary antibody. DAPI was used for nuclear counterstaining and images were captured under a confocal microscope (Carl Zeiss, Germany).

### Statistical analysis

All quantitative data are reported as the mean ± standard error of the mean (SEM). Multiple group comparisons were performed by one-way ANOVA using GraphPad Prism 6 software. P values<0.05 were considered statistically significant.

## Supplementary Material

Supplementary Figure 1

Supplementary Table 1

## References

[1] Bron AJ, de Paiva CS, Chauhan SK, Bonini S, Gabison EE, Jain S, Knop E, Markoulli M, Ogawa Y, Perez V, Uchino Y, Yokoi N, Zoukhri D. TFOS DEWS II pathophysiology report. Ocul Surf. 2017; 15:438–510. 10.1016/j.jtos.2017.05.011 28736340

[2] Kokado M, Miyajima M, Okada Y, Ichikawa K, Yamanaka O, Liu CY, Kao WW, Shou W, Saika S. Lack of plakoglobin impairs integrity and wound healing in corneal epithelium in mice. Lab Invest. 2018; 98:1375–83. 10.1038/s41374-018-0082-z 29802338

[3] Katzman LR, Jeng BH. Management strategies for persistent epithelial defects of the cornea. Saudi J Ophthalmol. 2014; 28:168–72. 10.1016/j.sjopt.2014.06.011 25278792 PMC4181455

[4] Wilson SE, Medeiros CS, Santhiago MR. Pathophysiology of corneal scarring in persistent epithelial defects after PRK and other corneal injuries. J Refract Surg. 2018; 34:59–64. 10.3928/1081597X-20171128-01 29315443 PMC5788463

[5] Samaeekia R, Rabiee B, Putra I, Shen X, Park YJ, Hematti P, Eslani M, Djalilian AR. Effect of human corneal mesenchymal stromal cell-derived exosomes on corneal epithelial wound healing. Invest Ophthalmol Vis Sci. 2018; 59:5194–200. 10.1167/iovs.18-24803 30372747 PMC6203220

[6] Li F, Zhao SZ. Mesenchymal stem cells: potential role in corneal wound repair and transplantation. World J Stem Cells. 2014; 6:296–304. 10.4252/wjsc.v6.i3.296 25126379 PMC4131271

[7] Ljubimov AV, Saghizadeh M. Progress in corneal wound healing. Prog Retin Eye Res. 2015; 49:17–45. 10.1016/j.preteyeres.2015.07.002 26197361 PMC4651844

[8] You HJ, Han SK. Cell therapy for wound healing. J Korean Med Sci. 2014; 29:311–19. 10.3346/jkms.2014.29.3.311 24616577 PMC3945123

[9] Saghizadeh M, Kramerov AA, Svendsen CN, Ljubimov AV. Concise review: stem cells for corneal wound healing. Stem Cells. 2017; 35:2105–14. 10.1002/stem.2667 28748596 PMC5637932

[10] Zomer HD, Vidane AS, Gonçalves NN, Ambrósio CE. Mesenchymal and induced pluripotent stem cells: general insights and clinical perspectives. Stem Cells Cloning. 2015; 8:125–34. 10.2147/SCCAA.S88036 26451119 PMC4592031

[11] Kern S, Eichler H, Stoeve J, Klüter H, Bieback K. Comparative analysis of mesenchymal stem cells from bone marrow, umbilical cord blood, or adipose tissue. Stem Cells. 2006; 24:1294–301. 10.1634/stemcells.2005-0342 16410387

[12] Strioga M, Viswanathan S, Darinskas A, Slaby O, Michalek J. Same or not the same? comparison of adipose tissue-derived versus bone marrow-derived mesenchymal stem and stromal cells. Stem Cells Dev. 2012; 21:2724–52. 10.1089/scd.2011.0722 22468918

[13] Zhang J, Guan J, Niu X, Hu G, Guo S, Li Q, Xie Z, Zhang C, Wang Y. Exosomes released from human induced pluripotent stem cells-derived MSCs facilitate cutaneous wound healing by promoting collagen synthesis and angiogenesis. J Transl Med. 2015; 13:49. 10.1186/s12967-015-0417-0 25638205 PMC4371881

[14] Hu GW, Li Q, Niu X, Hu B, Liu J, Zhou SM, Guo SC, Lang HL, Zhang CQ, Wang Y, Deng ZF. Exosomes secreted by human-induced pluripotent stem cell-derived mesenchymal stem cells attenuate limb ischemia by promoting angiogenesis in mice. Stem Cell Res Ther. 2015; 6:10. 10.1186/scrt546 26268554 PMC4533800

[15] Chien Y, Liao YW, Liu DM, Lin HL, Chen SJ, Chen HL, Peng CH, Liang CM, Mou CY, Chiou SH. Corneal repair by human corneal keratocyte-reprogrammed iPSCs and amphiphatic carboxymethyl-hexanoyl chitosan hydrogel. Biomaterials. 2012; 33:8003–16. 10.1016/j.biomaterials.2012.07.029 22858046

[16] Zhang J, Liu X, Li H, Chen C, Hu B, Niu X, Li Q, Zhao B, Xie Z, Wang Y. Exosomes/tricalcium phosphate combination scaffolds can enhance bone regeneration by activating the PI3K/Akt signaling pathway. Stem Cell Res Ther. 2016; 7:136. 10.1186/s13287-016-0391-3 27650895 PMC5028974

[17] Zemljic M, Pejkovic B, Krajnc I, Kocbek L. Modern stem cell therapy: approach to disease. Wien Klin Wochenschr. 2015 (Suppl 5); 127:S199–203. 10.1007/s00508-015-0903-7 26659705

[18] Ang AY, Chan CC, Biber JM, Holland EJ. Ocular surface stem cell transplantation rejection: incidence, characteristics, and outcomes. Cornea. 2013; 32:229–36. 10.1097/ICO.0b013e318255eac4 22668584

[19] Toh WS, Lai RC, Hui JH, Lim SK. MSC exosome as a cell-free MSC therapy for cartilage regeneration: implications for osteoarthritis treatment. Semin Cell Dev Biol. 2017; 67:56–64. 10.1016/j.semcdb.2016.11.008 27871993

[20] Azmi AS, Bao B, Sarkar FH. Exosomes in cancer development, metastasis, and drug resistance: a comprehensive review. Cancer Metastasis Rev. 2013; 32:623–42. 10.1007/s10555-013-9441-9 23709120 PMC3843988

[21] Gorecka J, Kostiuk V, Fereydooni A, Gonzalez L, Luo J, Dash B, Isaji T, Ono S, Liu S, Lee SR, Xu J, Liu J, Taniguchi R, et al. The potential and limitations of induced pluripotent stem cells to achieve wound healing. Stem Cell Res Ther. 2019; 10:87. 10.1186/s13287-019-1185-1 30867069 PMC6416973

[22] Bastos-Amador P, Royo F, Gonzalez E, Conde-Vancells J, Palomo-Diez L, Borras FE, Falcon-Perez JM. Proteomic analysis of microvesicles from plasma of healthy donors reveals high individual variability. J Proteomics. 2012; 75:3574–84. 10.1016/j.jprot.2012.03.054 22516433

[23] Carney EF. Chronic kidney disease: key role of exosomes in albumin-induced inflammation. Nat Rev Nephrol. 2018; 14:142. 10.1038/nrneph.2018.6 29355175

[24] Conde-Vancells J, Rodriguez-Suarez E, Embade N, Gil D, Matthiesen R, Valle M, Elortza F, Lu SC, Mato JM, Falcon-Perez JM. Characterization and comprehensive proteome profiling of exosomes secreted by hepatocytes. J Proteome Res. 2008; 7:5157–66. 10.1021/pr8004887 19367702 PMC2696236

[25] Danesh A, Inglis HC, Jackman RP, Wu S, Deng X, Muench MO, Heitman JW, Norris PJ. Exosomes from red blood cell units bind to monocytes and induce proinflammatory cytokines, boosting t-cell responses in vitro. Blood. 2014; 123:687–96. 10.1182/blood-2013-10-530469 24335232 PMC3907755

[26] Mathivanan S, Lim JW, Tauro BJ, Ji H, Moritz RL, Simpson RJ. Proteomics analysis of A33 immunoaffinity-purified exosomes released from the human colon tumor cell line LIM1215 reveals a tissue-specific protein signature. Mol Cell Proteomics. 2010; 9:197–208. 10.1074/mcp.M900152-MCP200 19837982 PMC2830834

[27] Wang L, Hu L, Zhou X, Xiong Z, Zhang C, Shehada HM, Hu B, Song J, Chen L. Exosomes secreted by human adipose mesenchymal stem cells promote scarless cutaneous repair by regulating extracellular matrix remodelling. Sci Rep. 2017; 7:13321. 10.1038/s41598-017-12919-x 29042658 PMC5645460

[28] Hu L, Wang J, Zhou X, Xiong Z, Zhao J, Yu R, Huang F, Zhang H, Chen L. Exosomes derived from human adipose mensenchymal stem cells accelerates cutaneous wound healing via optimizing the characteristics of fibroblasts. Sci Rep. 2016; 6:32993. 10.1038/srep32993 27615560 PMC5018733

[29] Aliotta JM, Pereira M, Wen S, Dooner MS, Del Tatto M, Papa E, Goldberg LR, Baird GL, Ventetuolo CE, Quesenberry PJ, Klinger JR. Exosomes induce and reverse monocrotaline-induced pulmonary hypertension in mice. Cardiovasc Res. 2016; 110:319–30. 10.1093/cvr/cvw054 26980205 PMC4872877

[30] Lai RC, Chen TS, Lim SK. Mesenchymal stem cell exosome: a novel stem cell-based therapy for cardiovascular disease. Regen Med. 2011; 6:481–92. 10.2217/rme.11.35 21749206

[31] Eberle I, Moslem M, Henschler R, Cantz T. Engineered MSCs from patient-specific iPS cells. Adv Biochem Eng Biotechnol. 2013; 130:1–17. 10.1007/10_2012_156 22915200

[32] Chandrasekher G, Pothula S, Maharaj G, Bazan HE. Differential effects of hepatocyte growth factor and keratinocyte growth factor on corneal epithelial cell cycle protein expression, cell survival, and growth. Mol Vis. 2014; 20:24–37. 24426773 PMC3888494

[33] Liu Y, Dong Y, Zhao L, Su L, Diao K, Mi X. TRIM59 overexpression correlates with poor prognosis and contributes to breast cancer progression through AKT signaling pathway. Mol Carcinog. 2018; 57:1792–802. 10.1002/mc.22897 30175868

[34] Nicoletti A, Vasta R, Venti V, Mostile G, Lo Fermo S, Patti F, Scillieri R, De Cicco D, Volanti P, Marziolo R, Maimone D, Fiore M, Ferrante M, Zappia M. The epidemiology of amyotrophic lateral sclerosis in the mount etna region: a possible pathogenic role of volcanogenic metals. Eur J Neurol. 2016; 23:964–72. 10.1111/ene.12973 26924209

[35] La Greca A, Solari C, Furmento V, Lombardi A, Biani MC, Aban C, Moro L, García M, Guberman AS, Sevlever GE, Miriuka SG, Luzzani C. Extracellular vesicles from pluripotent stem cell-derived mesenchymal stem cells acquire a stromal modulatory proteomic pattern during differentiation. Exp Mol Med. 2018; 50:119. 10.1038/s12276-018-0142-x 30201949 PMC6131549

[36] Ahdi A, Rabbani H, Vard A. A hybrid method for 3D mosaicing of OCT images of macula and optic nerve head. Comput Biol Med. 2017; 91:277–90. 10.1016/j.compbiomed.2017.10.031 29102825

[37] Akashi A, Kanamori A, Nakamura M, Fujihara M, Yamada Y, Negi A. Comparative assessment for the ability of cirrus, RTVue, and 3D-OCT to diagnose glaucoma. Invest Ophthalmol Vis Sci. 2013; 54:4478–84. 10.1167/iovs.12-11268 23737470

[38] Yoo YS, Whang WJ, Kim HS, Joo CK, Yoon G. Preoperative biometric measurements with anterior segment optical coherence tomography and prediction of postoperative intraocular lens position. Medicine (Baltimore). 2019; 98:e18026. 10.1097/MD.0000000000018026 31852065 PMC6922509

[39] Mort RL, Ramaesh T, Kleinjan DA, Morley SD, West JD. Mosaic analysis of stem cell function and wound healing in the mouse corneal epithelium. BMC Dev Biol. 2009; 9:4. 10.1186/1471-213X-9-4 19128502 PMC2639382

[40] Vizoso FJ, Eiro N, Cid S, Schneider J, Perez-Fernandez R. Mesenchymal stem cell secretome: toward cell-free therapeutic strategies in regenerative medicine. Int J Mol Sci. 2017; 18:1852. 10.3390/ijms18091852 28841158 PMC5618501

[41] Shen T, Zheng QQ, Shen J, Li QS, Song XH, Luo HB, Hong CY, Yao K. Effects of adipose-derived mesenchymal stem cell exosomes on corneal stromal fibroblast viability and extracellular matrix synthesis. Chin Med J (Engl). 2018; 131:704–12. 10.4103/0366-6999.226889 29521294 PMC5865317

[42] Zhang B, Wang M, Gong A, Zhang X, Wu X, Zhu Y, Shi H, Wu L, Zhu W, Qian H, Xu W. HucMSC-exosome mediated-Wnt4 signaling is required for cutaneous wound healing. Stem Cells. 2015; 33:2158–68. 10.1002/stem.1771 24964196

[43] Kalra H, Adda CG, Liem M, Ang CS, Mechler A, Simpson RJ, Hulett MD, Mathivanan S. Comparative proteomics evaluation of plasma exosome isolation techniques and assessment of the stability of exosomes in normal human blood plasma. Proteomics. 2013; 13:3354–64. 10.1002/pmic.201300282 24115447

[44] Lötvall J, Hill AF, Hochberg F, Buzás EI, Di Vizio D, Gardiner C, Gho YS, Kurochkin IV, Mathivanan S, Quesenberry P, Sahoo S, Tahara H, Wauben MH, et al. Minimal experimental requirements for definition of extracellular vesicles and their functions: a position statement from the international society for extracellular vesicles. J Extracell Vesicles. 2014; 3:26913. 10.3402/jev.v3.26913 25536934 PMC4275645

[45] Shao H, Im H, Castro CM, Breakefield X, Weissleder R, Lee H. New technologies for analysis of extracellular vesicles. Chem Rev. 2018; 118:1917–50. 10.1021/acs.chemrev.7b00534 29384376 PMC6029891

[46] Burger D, Viñas JL, Akbari S, Dehak H, Knoll W, Gutsol A, Carter A, Touyz RM, Allan DS, Burns KD. Human endothelial colony-forming cells protect against acute kidney injury: role of exosomes. Am J Pathol. 2015; 185:2309–23. 10.1016/j.ajpath.2015.04.010 26073035

[47] Doeppner TR, Herz J, Görgens A, Schlechter J, Ludwig AK, Radtke S, de Miroschedji K, Horn PA, Giebel B, Hermann DM. Extracellular vesicles improve post-stroke neuroregeneration and prevent postischemic immunosuppression. Stem Cells Transl Med. 2015; 4:1131–43. 10.5966/sctm.2015-0078 26339036 PMC4572905

