# Supplementary material for: Comparison of exosomes derived from induced pluripotent stem cells and mesenchymal stem cells as therapeutic nanoparticles for treatment of corneal epithelial defects
Source: Aging (Albany NY). 2020 Oct 13;12(19):19546–62. doi: 10.18632/aging.103904 (PMC7732275; doi:10.18632/aging.103904)
Supplement: Supplementary Figure 1 [file aging-12-103904-s001..pdf]

SUPPLEMENTARY FIGURE

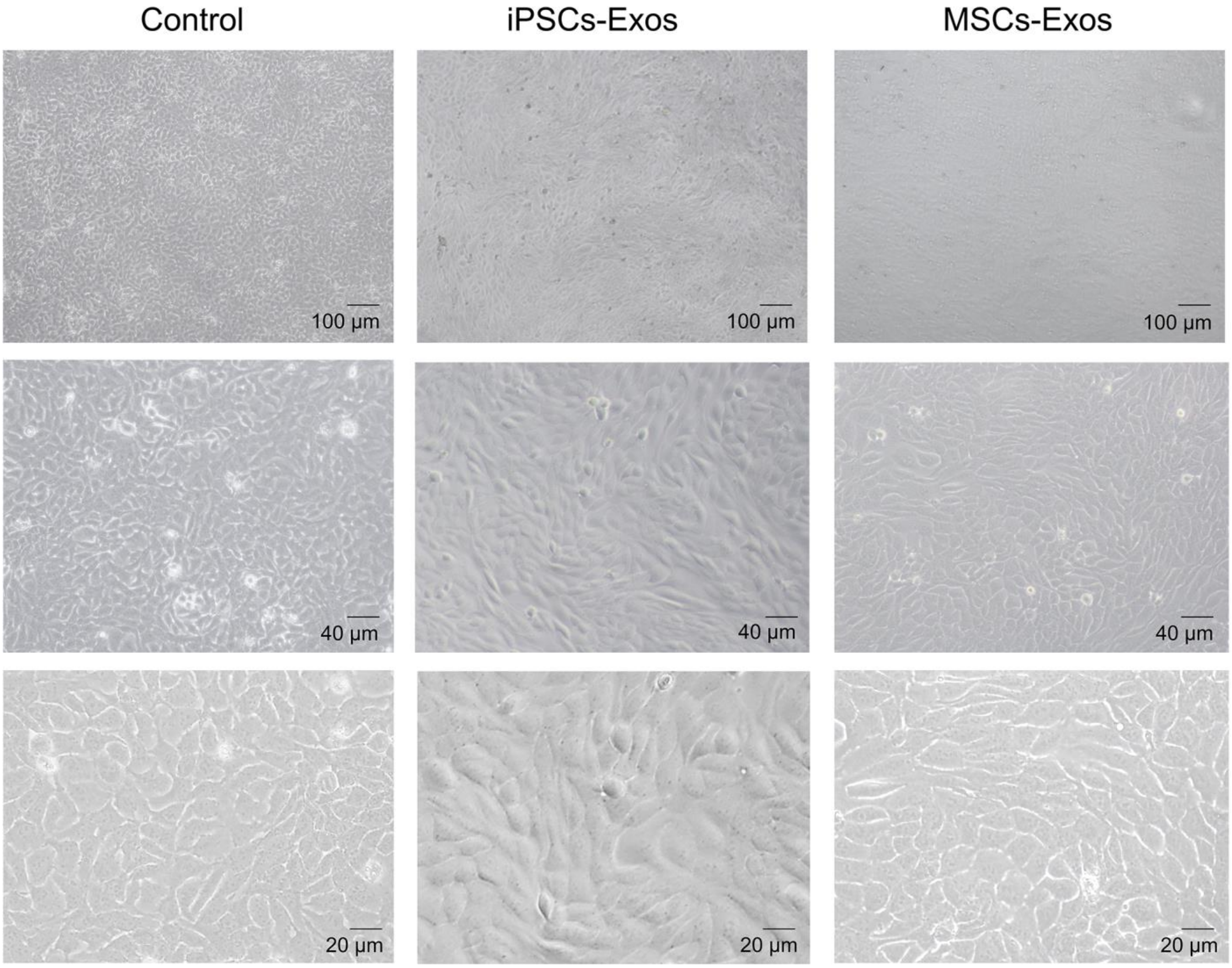

Supplememtary Figure 1. Cell morphology of HCECs. After HECEs was stimulated with iPSCs/MSCs-Exos or vehicle for 48 h, cell morphology was taken by microscope. HECEs in control group showed atrophy and increased apoptotic cells, while those with iPSCs/MSCs-Exos showed a much fuller rounded shape.
