# Supplementary material for: Comparison of exosomes derived from induced pluripotent stem cells and mesenchymal stem cells as therapeutic nanoparticles for treatment of corneal epithelial defects
Source: Aging (Albany NY). 2020 Oct 13;12(19):19546–62. doi: 10.18632/aging.103904 (PMC7732275; doi:10.18632/aging.103904)
Supplement: Supplementary Table 1 [file aging-12-103904-s002..pdf]

SUPPLEMENTARY TABLES

Supplementary Table 1. Primer sequences used for qRT-PCR.

Gene	Primer sequence
Cyclin A	F: AACTTCAGCTTGTGGGCACT; R: CTGGTGGGTTGAGGAGAGAA
Cyclin E	F: CCATCCTTCTCCACCAAAGA; R: AGCACCTTCCATAGCAGCAT
CDK2	F: CCAGGAGTTACTTCTATGCCTGA; R: TTCATCCAGGGGAGGTACAAC
PCNA	F: GCCAGAGCTCTTCCCTTACG; R: TAGCTGGTTTCGGCTTCAGG
GAPDH	F: AAGAAGGTGGTGAAGCAGGC; R: TCCACCACCCTGTTGCTGTA
